# Response of Two Dominant Boreal Freshwater Wetland Plants to Manipulated Warming and Altered Precipitation

**DOI:** 10.1371/journal.pone.0104454

**Published:** 2014-08-08

**Authors:** Yuanchun Zou, Guoping Wang, Michael Grace, Xiaonan Lou, Xiaofei Yu, Xianguo Lu

**Affiliations:** 1 Key Lab of Wetland Ecology and Environment, Northeast Institute of Geography and Agroecology, Chinese Academy of Sciences, Changchun, China; 2 Water Studies Centre and School of Chemistry, Monash University, Clayton, Australia; University of Saskatchewan, Canada

## Abstract

This study characterized the morphological and photosynthetic responses of two wetland plant species when they were subject to 2–6°C fluctuations in growth temperature and ±50% of precipitation, in order to predict the evolution of natural wetlands in Sanjiang Plain of North-eastern China. We investigated the morphological and photosynthetic responses of two dominant and competitive boreal freshwater wetland plants in Northeastern China to manipulation of warming (ambient, +2.0°C, +4.0°C, +6.0°C) and altered precipitation (−50%, ambient, +50%) simultaneously by incubating the plants from seedling to senescence within climate-controlled environmental chambers. Post-harvest, secondary growth of *C. angustifolia* was observed to explore intergenerational effects. The results indicated that *C. angustifolia* demonstrated a greater acclimated capacity than *G. spiculosa* to respond to climate change due to higher resistance to temperature and precipitation manipulations. The accumulated effect on aboveground biomass of post-harvest secondary growth of *C. angustifolia* was significant. These results explain the expansion of *C. angustifolia* during last 40 years and indicate the further expansion in natural boreal wetlands under a warmer and wetter future. Stability of the natural surface water table is critical for the conservation and restoration of *G. spiculosa* populations reacting to encroachment stress from *C. angustifolia* expansion.

## Introduction

Wetlands are among the most important terrestrial carbon pools and play an important role in global carbon cycling [1], [2]. Boreal wetlands comprise about half of the total global wetland area, store about one third of the world's organic carbon in the form of living or partially decayed vegetation and soil organic carbon and contribute 34% of global atmospheric methane flux [3], [4]. Due to their large and dynamic carbon pool, boreal wetlands may not be only significantly impacted by climate change, but may also provide negative (e.g. increasing organic carbon sink) or positive feedback (e.g. increasing greenhouse gas emissions) mechanisms to anthropogenic climate change [5], [6] and mitigate climate warming overall.

Boreal wetlands are considered particularly vulnerable to climate change because they depend on specific climatic conditions with low temperature and high water availability [7], [8]. To quantify anticipated climate effects on boreal wetland ecosystem processes, field experiments simulating climate change have been undertaken in North America and northern Europe [2], [5], [9], and these studies suggested that both the performance of a specific plant and the compositions of plant communities will change in different directions and at different magnitudes as a response to warming and/or changes in the depth of the surface water table that usually connected with temperature and precipitation change. Despite these efforts, species-specific eco-physiological responses of wetland plants to changes in temperature and precipitation, and especially the interactive effects of these factors, remain unclear. Simulations provide a mechanism for exploring the importance of these temperature-precipitation interactions on wetland plants under future climate conditions [10]. Multifactor experimental manipulations of temperature and precipitation in the laboratory can complement field studies by enabling controlled adjustment of covarying climate variables based on the historical data to generate relatively realistic climate change experimental scenarios [11]. The responses then could be scaled up to the level of the ecosystem when the initial structure of the wetland and the environmental driving variables are consequently well coupled.

As typical dominant boreal freshwater wetland plants in Northeast China, Siberia and Far East Russia, *Calamagrostis angustifolia* and *Glyceria spiculosa* both belong to the family Poaceae, and are often distributed as large, dense clumps in palustrine, riverine and lacustrine wetlands [12], [13]. Generally, *C. angustifolia* covers saturated microhabitats while *G. spiculosa* covers the flooded microhabitats with greater surface water depth. Both plants play important ecological functions in the wetlands as primary producers for food webs, shelter for some invertebrates and major organic carbon sources for carbon sequestration. In recent years, with climate warming and surface water deficiency in some wetlands of the Sanjiang Plain, the microhabitats once covered by *G. spiculosa* have been almost replaced by *C. angustifolia* due to the drawdown of surface water depth, caused either by climate change or anthropogenic activities such as drainage.

In this paper, we simultaneously considered two major factors (temperature and precipitation) involved in climate change effects on boreal wetland ecosystem functioning. The two plant species, *C. angustifolia* and *G. spiculosa*, were incubated from seedling to senescence in environmental chambers under manipulated warming and altered precipitation conditions. The objectives of this study were: (1) to characterize the morphological and photosynthetic responses of the two plants to 2–6°C warming and ±50% of precipitation changes; and (2) to analyze the resistance and acclimation of the two plants and predict their potential distribution in the future under the projected changed climate conditions in the Sanjiang Plain wetlands of Northeast China.

## Materials and Methods

### Study site and target species

The study site is located in Xingkai Lake (Khanka) lacustrine wetlands of Sanjiang Plain, the largest expanse of freshwater wetlands in China. Xingkai Lake is the largest lake in Northeast Asia and a transboundary lake shared by China and Russia. According to calculations based on the 54 years of local meteorological records, the annual mean precipitation is 561 mm and the temperature ranges from −39°C in January to 36°C in July with the annual mean of 3.5°C for 1957–2010. According to a linear fitting of local meteorological records in Xingkai Lake lacustrine wetlands (Annual temperature  =  −52.55 + 0.028*year, *r*>0.99, *p*<0.0001; unpublished), there is a significant warming trend of 0.28°C per decade in Xingkai Lake over the past half century. This is even greater than the average level of 0.22°C per decade for the whole country [14].

The target plant species for the three-month incubation experiments were collected *in situ* from *Calamagrostis angustifolia* (45°20′59" N, 132°18′52" E) and *Glyceria spiculosa* (45°21′49" N, 132°19′26" E) communities within Xingkai Lake lacustrine wetlands. Representative and homogeneous individuals of *C. angustifolia* and *G. spiculosa* were excavated with intact roots and soils using a planting shovel in May of 2011 and 2012, respectively, when the first new shoots were apparent in springtime. The soil types were silty clay meadow marsh soil and humus marsh soil, respectively. The seedlings and soils with the depth of approximate 20 cm were cut into small blocks with rhizome based, transported to the laboratory in Northeast Institute of Geography and Agroecology within two days, and transplanted into incubation pots with 10-cm diameter and 15-cm depth with 4–5 seedlings in each pot. To minimize loss of vitality during transplantation, these potted seedlings were first incubated in a greenhouse for one month to germinate new ramets before placement into environmental chambers. The temperature in the greenhouse was set as the average air temperature of June in Xingkai Lake lacustrine wetlands. All the field studies got the permission from administrative authority of Xingkai Lake National Nature Reserve and did not involve any endangered or protected species.

### Experimental design

The design between the warming and the precipitation was a two-way randomized block experiment. Four environmental chambers were deployed to control the temperature. Within each chamber, three plastic container (35 cm × 25 cm × 10 cm) were used to control water level fluctuations caused by precipitation manipulation. Each container contained three replicated pots. Before incubation, any pot growing approximate ten vigorous seedlings with similar performance (about 20 cm and 15 cm heights for *C. angustifolia* and *G. spiculosa*, respectively) was selected and randomly separated into 4 groups corresponding to the 4 temperature regimes. The ambient temperature was set as the local monthly average air temperatures in July, August and September, respectively fitted. The ambient temperature was adjusted five times per day based on the local monthly (July, August and September) average air temperatures over the time periods 1:00–4:00, 4:00–10:00, 10:00–16:00, 16:00–19:00 and 19:00–1:00. Table 1 provides the temperature and illuminance values for the plant incubations in the environmental chambers. The illuminance values were based on the field observation. These pots were exchange their positions in the chambers daily to keep the temperature and light intensity similar for a given pot. The warming scenarios were then set according to the ambient temperature regime with +2°C, +4°C and +6°C added for each interval. The lights were plant growth fluorescent lamps that are recognized as suitable for plants' growth [15].

**Table 1:** Temperature and illuminance control for plants' incubation in the environmental chambers.

Time period		0:00–7:00	7:00–11:00	11:00–14:00	14:00–16:00	16:00–24:00
Illuminance (lux)		0	10000	20000	15000	5000
Temperature	July	16	20	28	24	17
(°C)	August	17	19	26	23	18
	September	11	14	19	17	12

The ambient water depths were set to mimic the natural conditions in Xingkai Lake wetlands, with no net water for *C. angustifolia* and 10 cm of water above the soil surface for *G. spiculosa*. The ambient precipitation regime was set according to the local monthly average precipitation. These seedlings were gently watered over one hour using sprinkler nozzles to simulate natural precipitation every 3 days with one tenth of the amount of monthly precipitation. The altered precipitations were set as -50% and +50% based on the ambient precipitation rates.

After the three month incubation in the environmental chambers and subsequent harvesting of all above-ground biomass, one *C. angustifolia* pot was randomly selected from the 3 replicates of each precipitation treatment container within each environmental chamber. Twelve pots were consequently selected for a secondary growth test to analyze the potential effect of climate change on the next generation of this plant species. These pots were incubated for one month in the same environmental chamber with the average temperature and precipitation in June of 17.4°C and 74.4 mm, respectively in Xingkai Lake wetlands.

### Response measurements

The morphological indicators (height, stem diameter and leaf area) were observed once per month using a meter stick, vernier caliper and portable area meter (LI-3000, Li-Cor Inc., USA) from seedling to senescence, with each indicator measured by 4 replicates for *C. angustifolia* and 3 replicates for *G. spiculosa*. The photosynthetic indicators, including leaf net photosynthetic rate (Pn), leaf stomatal conductance (Gs) and leaf transpiration rate (Ts) were measured by a portable photosynthesis system (LI-6400, Li-Cor, Inc., USA) during the peak growth phase (around August), with each indicator measured by 4 replicates for *C. angustifolia* and 3 replicates for *G. spiculosa*. The chlorophyll content was determined by homogenizing and extracting the leaf tissue in 80% acetone until the green colour turn white completely, then measuring the optical absorbance at 663 and 645 nm for chlorophyll a and b, respectively using a spectrophotometer (UV-2500, Shimadzu, Japan), and calculating concentrations using the specific absorption coefficients [16], [17]. The reported leaf chlorophyll content was the sum of chlorophyll a and b, with 3 replicates both for *C. angustifolia* and *G. spiculosa*.

Above-ground biomass was measured after plant senescence by drying to constant weight at 80°C. The total nitrogen content of harvested leaves was determined using a continuous flow analyzer (SAN^++^, SKALAR, the Netherlands) after digesting 0.3000 g of homogeneous, milled material with H_2_SO_4_/H_2_O_2_, with 3 replicates both for *C. angustifolia* and *G. spiculosa*. The below-ground biomass was not measured as it is a mixture of newly developed rhizomes/roots within that growing season and the rhizomes/roots from previous growing seasons. Older material usually accounts for most of the total below-ground biomass [18].

The response measurements for the secondary growth test of *C. angustifolia* were conducted as described above. The measured indicators were height, stem diameter above the soil, leaf area of each leaf on the plant based on measuring a suitable number of leaves, Pn, Gs, Ts and above-ground biomass, with each indicator measured by 3 replicates.

### Statistical analyses

Statistical analyses were performed using SPSS Statistics 21.0 (SPSS Inc., USA). The main and the interaction effects of temperature and precipitation on the two plants' morphological indicators were compared by repeated measures analysis of variance (ANOVA). For repeated measures ANOVA, Mauchly's Test of Sphericity was first examined and the Greenhouse-Geisser adjustment was adopted when the null hypothesis was rejected. The main and the interaction effects of temperature and precipitation on the two plants' photosynthetic indicators, leaf chlorophyll contents during the phase of peak growth, the above-ground biomasses and leaf nitrogen contents after senescence were compared by two-way ANOVA. One-way ANOVA was performed to compare the differences of above-ground biomasses of *C. angustifolia* and *G. spiculosa*. Differences in morphology, photosynthesis and above-ground biomass between different secondary grown *C. angustifolia* after the temperature and precipitation manipulation for one month were also compared using one-way ANOVA. The least significant difference (LSD) was used respectively to perform *post hoc* multiple comparisons when equal variances were assumed; otherwise, Tamhane's T2 method was used.

## Results

### Morphological indicators

Repeated-measures ANOVA of morphological indicators showed that the interaction effects of temperature and precipitation on the height of *C. angustifolia* and *G. spiculosa* were both significant (*F* = 2.025, *p* = 0.044; *F* = 2.520, *p* = 0.033, respectively). For both species, main effects of temperature or precipitation, and any interaction effects, on basal stem diameter were all insignificant. The main effects of temperature on leaf area of *C. angustifolia* and the effect of precipitation on that of *G. spiculosa* were both significant (*F* = 2.449, *p* = 0.047; *F* = 3.271, *p* = 0.031, respectively) (Table 2).

**Table 2:** Repeated-measures analysis of variance (ANOVA) of morphological indicators for *Calamagrostis angustifolia* and *Glyceria spiculosa*.

Variable		Month		Month × Temperature		Month × Precipitation		Month × Temperature × Precipitation	
		*F*	*p*	*F*	*p*	*F*	*p*	*F*	*p*
*C. angustifolia*	Height	30.864	**<0.001**	0.910	0.482	2.611	0.052	2.025	**0.044**
	Basal stem diameter	0.064	0.860	0.538	0.702	0.343	0.764	0.513	0.835
	Leaf area	151.032	**<0.001**	2.449	**0.047**	0.271	0.857	0.922	0.517
*G. spiculosa*	Height	111.065	**<0.001**	1.038	0.412	2.203	0.116	2.520	**0.033**
	Basal stem diameter	3.165	0.051	0.412	0.867	1.426	0.240	0.839	0.612
	Leaf area	61.079	**<0.001**	1.739	0.155	3.271	**0.031**	1.363	0.239

Significant effects (*p*<0.05) are shown in bold. When the highest order of interaction effect is significant, the significant main effect is not in bold.

**Figure 1:**
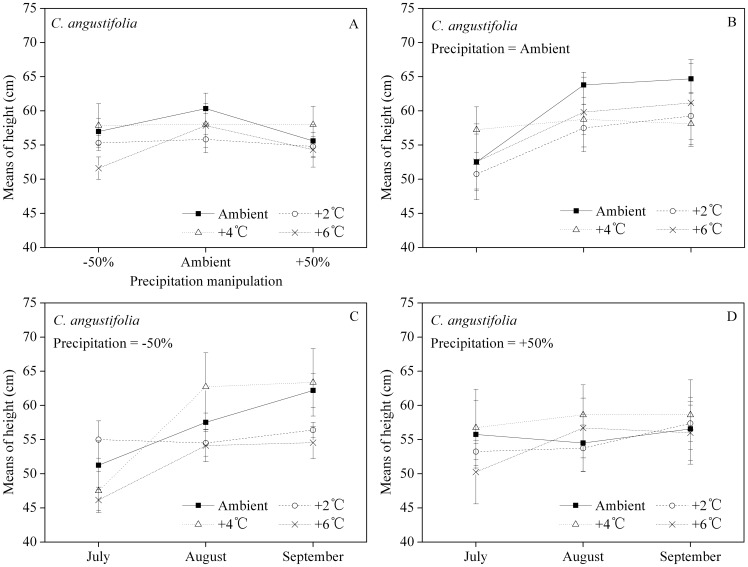
Interaction of temperature and precipitation manipulations on plant height (A) of *Calamagrostis angustifolia*, and the monthly estimated marginal means of height of *C. angustifolia* for different temperature treatments when precipitation was manipulated at ambient (B), −50% (C) and +50% (D) levels, respectively. The error bars represent means ± 1 standard error.

*C. angustifolia*'s height increased from July to September. The estimated marginal means (as the average of the results for each month) of the height suggested that the greatest heights were observed in the ambient precipitation for each warming manipulation (Fig. 1A). Under ambient precipitation conditions (Fig. 1B), the greatest height occurred at ambient temperature (except for the early growth of July), while the lowest height was found in the +2°C manipulation (except in September). When the precipitation decreased by 50% from ambient (Fig. 1C), the greatest height occurred in the +4°C manipulation (except for the early growth of July), while the lowest height was in the +6°C manipulation. When the precipitation increased by 50% from ambient, the greatest height occurred in the +4°C manipulation, while the other three treatments produced plants of comparable height (Fig. 1D).

**Figure 2:**
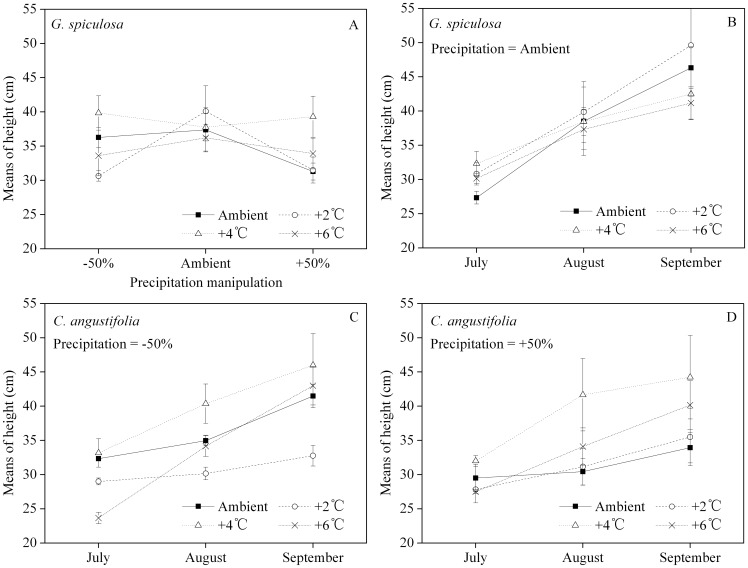
Interaction of temperature and precipitation manipulations on plant height (A) of *Glyceria spiculosa*, and the monthly estimated marginal means of height of *G. spiculosa* for different temperature treatments when the precipitation was manipulated at ambient (B), −50% (C) and +50% (D) levels, respectively. The error bars represent means ± 1 standard error.

*G. spiculosa*'s height also increased throughout the incubation (Fig. 2B–D). The estimated marginal means of the height suggested that ambient precipitation and 2–4°C of warming could support greater plant height (Fig. 2A). In the ambient precipitation conditions, there were no significant differences in height between the different temperature treatments (Fig. 2B). For both the −50% and +50% precipitation treatments, the greatest height occurred in the +4°C manipulation (Fig. 2C, D).

**Figure 3:**
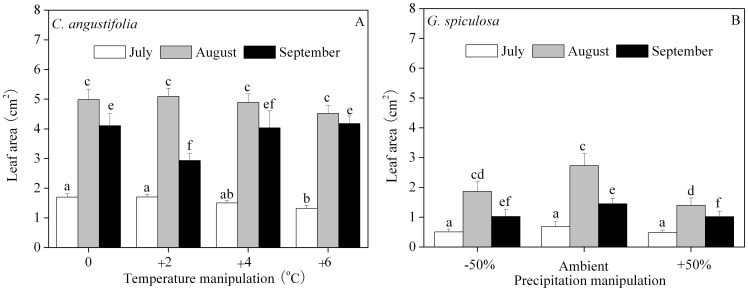
Leaf area of *C. angustifolia* (A) and *G. spiculosa* (B) monthly. For each month, different letters over the bars indicate significant differences (*p*<0.05) between temperature manipulations for *C. angustifolia* and precipitation manipulations for *G. spiculosa*. The letters ‘a’ and ‘b’ are for the July results, ‘c’ and ‘d’ for the August results, and ‘e’ and ‘f’ for the September results. The error bars represent means ± 1 standard error.

The leaf area of the two species increased first and then decreased throughout the incubation. For *C. angustifolia*, the leaf area with the +6°C manipulation was smaller than other manipulations in July. There was no significant difference in leaf area between the ambient and the +2°C manipulation in July. In September, the leaf area with the +4°C manipulation was not significantly different from either the ambient or the +6°C manipulation. There was no significant difference in leaf area with different temperature manipulations in August (Fig. 3A). For *G. spiculosa*, there was no significant difference between ambient and the −50% precipitation treatments in any of the three months, while the leaf area with the 50% precipitation treatment was smaller than other treatments in August and September (Fig. 3B).

### Photosynthetic indicators and leaf chlorophyll content

Two-way ANOVA of photosynthetic indicators and leaf chlorophyll content showed that the interaction effects of temperature and precipitation on these two species were both significant, except for the net photosynthetic rate (Pn) for *G. spiculosa*, which was only significantly affected by precipitation (Table 3).

**Table 3:** Two-way ANOVA of photosynthetic indicators and leaf chlorophyll content for *C. angustifolia* and *G. spiculosa* during the phase of peak growth, and above-ground biomass and leaf total nitrogen content after harvest.

Variable		Temperature		Precipitation		Temperature × Precipitation	
		*F*	*p*	*F*	*p*	*F*	*p*
*C. angustifolia*	Pn	9.878	**<0.001**	7.516	**0.002**	4.974	**0.001**
	Gs	3.691	**0.021**	8.639	**0.001**	6.825	**<0.001**
	Ts	4.410	**0.010**	3.013	0.062	9.208	**<0.001**
	Chlorophyll	0.966	0.419	0.765	0.473	2.545	**0.037**
	Aboveground biomass	4.512	**0.012**	2.349	0.117	0.842	0.550
	Leaf total nitrogen	7.125	**0.001**	3.642	0.052	0.524	0.784
*G. spiculosa*	Pn	1.663	0.201	11.361	**<0.001**	2.244	0.073
	Gs	17.989	**<0.001**	1.966	0.162	3.400	**0.014**
	Ts	5.091	**0.007**	2.883	0.075	3.170	**0.020**
	Chlorophyll	4.920	**0.008**	7.153	**0.004**	2.322	**0.046**
	Aboveground biomass	4.156	**0.017**	13.637	**<0.001**	1.002	0.447
	Leaf total nitrogen	2.737	0.066	1.134	0.338	2.394	0.059

Significant effects (*p*<0.05) are shown in bold. When the highest order of interaction effect is significant, the significant main effect is not in bold.

**Figure 4:**
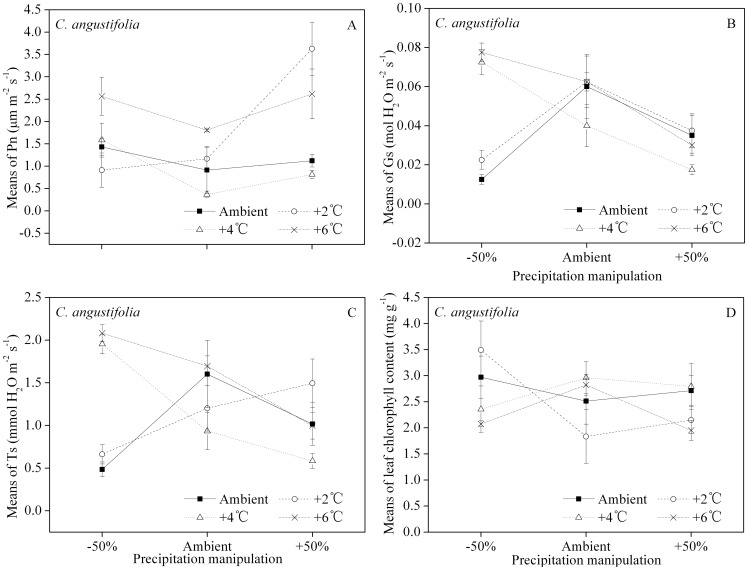
Interactions of temperature and precipitation manipulations on *C. angustifolia*'s leaf net photosynthetic rate (Pn, A), leaf stomatal conductance (Gs, B), leaf transpiration rate (Ts, C) and leaf chlorophyll content (D), respectively. The error bars represent means ± 1 standard error.

For *C. angustifolia*, smaller Pn occurred under ambient precipitation except for the +2°C manipulation, and greater Pn was measured in the +6°C manipulation with the exception of the +50% precipitation treatment (Fig. 4A). The leaf stomatal conductance, Gs, decreased with the enhanced precipitation for the +4°C and +6°C manipulations (Fig. 4B). The greatest leaf transpiration rates, Ts, were found in the +4°C and +6°C manipulations under −50% precipitation (Fig. 4C). Ts rates decreased in both these temperature treatments with increasing precipitation. There was no systematic pattern in Ts at ambient temperature but the +2°C manipulation showed Ts increased with increasing precipitation. There were no consistent trends in leaf chlorophyll content with temperature or precipitation treatments (Fig. 4C) but all concentrations fell within the range 1.83–3.49 mg/g.

**Figure 5:**
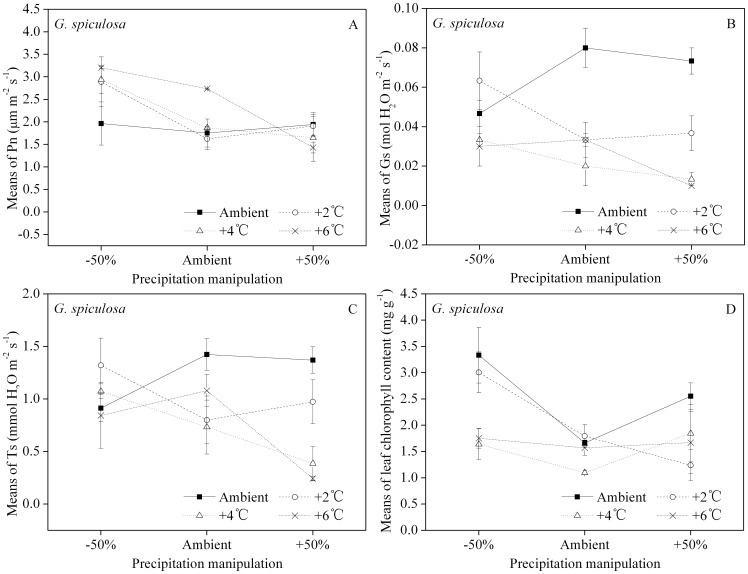
Main effect of precipitation manipulation on *G. spiculosa*'s leaf net photosynthetic rate (Pn, A), interactions of temperature and precipitation manipulations on leaf stomatal conductance (Gs, B), leaf transpiration rate (Ts, C) and leaf chlorophyll content (D), respectively. Different letters shared by the bars indicate significant differences (*p*<0.05) between precipitation treatments. The error bars represent means ± 1 standard error.

For *G. spiculosa*, Pn decreased with precipitation (Fig. 5A). With the exception of the +2°C treatment under the −50% precipitation regimen, the highest Gs results were found under ambient temperature (Fig. 5B). There was no consistent response in Gs to increased precipitation over the temperature treatments. There was no consistent trend in Ts across either temperature or precipitation treatments (Fig. 5C). All temperature treatments showed a decrease in leaf chlorophyll content between −50% and ambient precipitation (Fig. 5D). Increasing precipitation to 50% above ambient resulted in no consistent trend in chlorophyll with increasing temperature regime.

### Above-ground biomass and leaf nitrogen content

**Figure 6:**
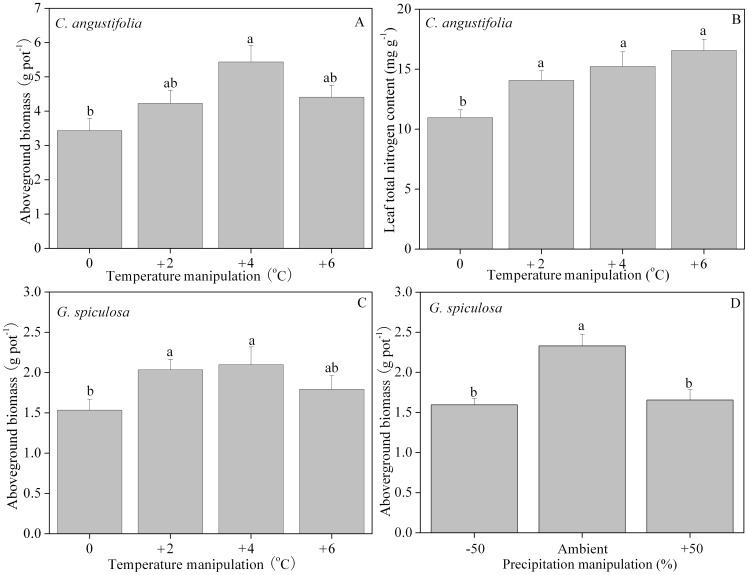
Aboveground biomass (A), leaf total nitrogen (B) of *C. angustifolia*, and aboveground biomass of *G. spiculosa* (C, D) after harvest. Different letters shared by the bars indicate significant differences (*p*<0.05) between temperature or precipitation manipulations. The error bars represent means ± 1 standard error.

Above-ground biomass of *C. angustifolia* was only significantly affected by temperature (*F* = 4.512, *p* = 0.012), while the effect of precipitation and the interaction effect were insignificant (Table 3). Biomass was at a maximum in the +4°C manipulation, increasing by 58% above that at ambient temperature (Fig. 6A). Leaf total nitrogen of *C. angustifolia* was also only significantly affected by temperature (*F* = 7.125, *p* = 0.001; Table 3). All three temperature increments resulted in statistically significant increases above ambient temperature, with a maximum value at +6°C, representing a 39% increase above ambient temperature (Fig. 6B).

Above-ground biomass of *G. spiculosa* was significantly affected both by temperature and precipitation (*F* = 4.156, *p* = 0.017; *F* = 13.637, *p*<0.0001, respectively), while the interaction effect was insignificant (Table 3). As noted with *C. angustifolia*, biomass was highest in the +4°C manipulation, with an increase of 37% above ambient temperature (Fig. 6C). Both increasing and decreasing precipitation had negative effects on above-ground biomass, with reductions of 29% and 32%, respectively compared to ambient precipitation (Fig. 6D). Any effects of temperature or precipitation, as well as their interaction effect, on leaf total nitrogen of *G. spiculosa* were insignificant (Table 3).

### Secondary growth of *C. angustifolia*

**Figure 7:**
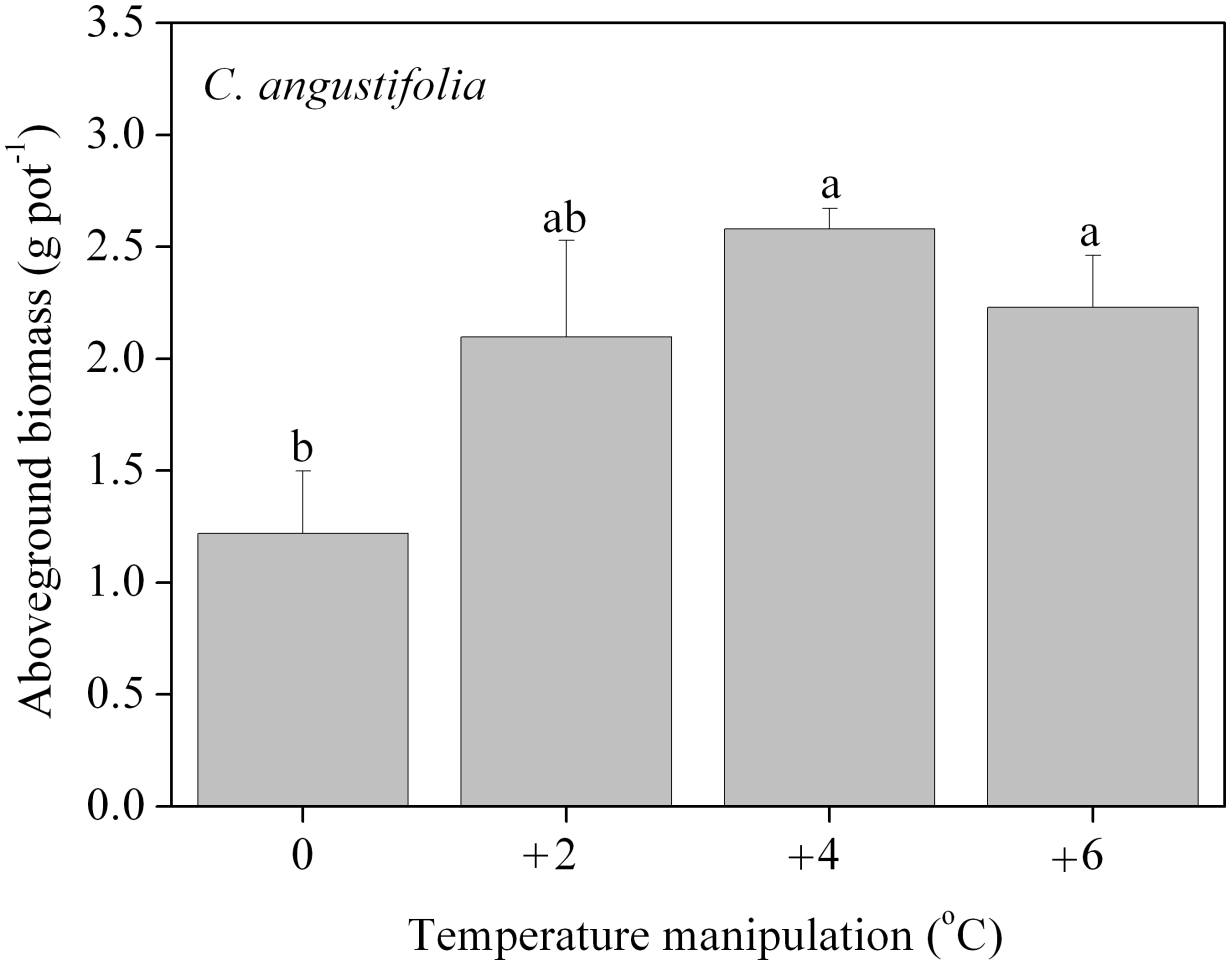
Aboveground biomass of *C. angustifolia* after secondary growth for one month. Different letters shared by the bars indicate significant differences (*p*<0.05) between the temperature manipulations. The error bars represent means ± 1 standard error.

One-way ANOVA results (Table 4) showed that only above-ground biomass displayed a significant difference with the incubation temperature treatment after one month's secondary growth (*F* = 4.096, *p* = 0.049); no other morphological or photosynthetic indicator showed significant differences (Table 4). Above-ground biomass of secondary growth was greatest in the +4°C manipulation, 1.11 times higher than the ambient temperature (Fig. 7).

**Table 4:** One-way ANOVA of aboveground biomass, morphological and photosynthetic indicators of *C. angustifolia* after secondary growing for one month.

ANOVA	Height	Basal stem diameter	Leaf area	Pn	Gs	Ts	Aboveground biomass
*F*	0.281	0.874	1.849	2.963	2.650	2.802	4.096
*p*	0.838	0.494	0.217	0.097	0.120	0.108	**0.049**

Significant effects (*p*<0.05) are shown in bold.

## Discussion

### Interaction effects of warming and precipitation alteration

Boreal wetlands are becoming increasingly vulnerable in a warming world [8]. Measuring responses of dominant plant species to warming and precipitation alteration is fundamental to assess and project the vulnerability of boreal wetlands under climate change. The interactions among multiple climate-change factors may cause responses in wetlands that may not be detected when focusing on a single factor, because climate change will include simultaneous changes in temperature and precipitation regimes [19], [20]. The above-ground biomass of *C. angustifolia* and *G. spiculosa* initially showed an increasing trend and then decreased with continued increments in mean monthly temperature. The +4°C temperature treatment produced the highest biomass (Fig. 6A, C), which is consistent with the single factor study by Breeuwer et al. [10] and multi-factor meta-analysis results from Lin et al. [21].

The monthly heights in different warming manipulations show intersecting trend lines (Fig. 1A, 2A), indicating that interaction effects of temperature and precipitation should be considered, especially for the +4°C manipulation. In the decreased precipitation treatment (−50%), greater plant heights were at ambient temperature and in the +4°C manipulation for *C. angustifolia* and *G. spiculosa* (Fig. 1C, 2C, respectively). With the exclusion of injury, height is one of the best indicators of an herbaceous plant's growth and competitiveness in light [22], [23]. Our results show that any study on species-specific or interspecific plant response to climate change should also include consideration of the accompanying precipitation regime.

A limited increase of temperature within the optimal range often enhances photosynthesis, depending on the species and its environmental acclimation [24], [25]. However, this expected enhancement of Pn can be moderated by concurrent water or drought stress on plants [26]. Our results show that Pn of *C. angustifolia* increased with temperature only in the driest (−50% precipitation) treatment, while ambient precipitation resulted in smaller Pn values in two of the three elevated temperatures (Fig. 4A), indicating that a change in precipitation would counter-intuitively limit the photosynthetic capacity of *C. angustifolia*. The Pn of *G. spiculosa* decreases with increasing precipitation (Fig. 5A), suggesting that soil water availability affects photosynthesis more than temperature variation [27]. Photosynthetic capacity is closely associated with, and approximately proportional to, leaf chlorophyll and nitrogen contents, and all three are favored by increased temperature [28], [29], [30]. Only the leaf nitrogen content of *C. angustifolia* increased with temperature (Table 3, Fig. 6B), while that of *G. spiculosa* was not significantly affected either by temperature or precipitation (Table 3). Although the leaf chlorophyll contents of two species were altered both by temperature and precipitation (Table 3), the temperature responses under different precipitation treatments differed between species (4D, 5D).

These differential, and even opposite, responses of two species to our manipulated temperature and precipitation treatments are consistent with the widely supported observations and modeling that show species respond individualistically to changes in environmental conditions [5].

### Species-specific responses to temperature and precipitation change

The effects of climate change on boreal wetland ecosystems ranges from regional, ecosystem, vegetation type, functional type to species [21], [31], [32], [33]. Our results suggest that within the same functional plant type, species-specific response was a significant determinant in height (Table 3, Fig. 1, 2), leaf area (Fig. 3), photosynthetic indicators, leaf chlorophyll contents (Table 3, Fig. 4, 5), and above-ground biomass (Table 3, Fig. 6).

The most marked species-specific difference was leaf area and above-ground biomass (Table 2, 3). Leaf area is only affected by temperature for *C. angustifolia* (Fig. 3A) and precipitation for *G. spiculosa* (Fig. 3B). Above-ground biomass of *C. angustifolia* is only affected by temperature (Fig. 6A), while both temperature and precipitation influence *G. spiculosa* (Fig. 6C, D). Given the direct relationship with net primary productivity, above-ground biomass is considered as one of the plant characteristics responding strongly to climate change [34]. The +2°C manipulation increased above-ground biomass 23% and 31% compared with ambient conditions for *C. angustifolia* (Fig. 6A) and *G. spiculosa* (Fig. 6C), respectively. According to the linear fitting of local meteorological records in Xingkai Lake lacustrine wetlands, a 2°C warming is likely to take place by the 2080s. Consequently, this species-specific difference in predicted biomass would affect carbon and nutrient budgets in boreal wetland ecosystems responding to future temperature change [31].

Previous species-specific comparisons under simulated climate change are mostly based on only one growth season, and the intergenerational response is not readily apparent [35], [36]. The significant difference in above-ground biomass of secondary growth (Fig. 7) with the different temperature treatments indicates that effects of climate change would continue to the next generation, because *C. angustifolia* mainly reproduces clonally by rhizomes [12]. These rhizomes would store a large amount of biomass below-ground, translocated from above-ground when the shoot is harvested. Greater biomass results in better performance during secondary growth [37]. Considering the biomass accumulation of biomass year by year, *C. angustifolia* is more acclimated to temperature and precipitation change. This acclimated capacity supports the continued presence of *C. angustifolia* under climate change and perhaps even expansion, because a competitor is not as evolutionarily successful in adapting to change and hence will decline leaving an ecological niche to be filled [31].

### Inter-specific interaction to temperature and precipitation change

Interspecies interactions play a key role in regulating the distribution and composition of plant communities. The impacts of inter-species interactions can be altered by external drivers such as climatic conditions or anthropogenic activities [38]. For well conserved boreal wetlands, climate change may affect wetland plant communities by directly limiting or fostering the performance of particular species or by altering species competition and abundance [10], [39].

As the two dominant species in Sanjiang Plain wetlands of Northeast China, *C. angustifolia* competes with *G. spiculosa* both in mesic and hydric habitats. Based on sequentially documented surveys of plant communities in a mesic habitat from 172 sampling plots from 1973 to 2003, the “importance values”, as the average of relative density, frequency and coverage which gives an overall estimate of the influence of importance of a plant species in the community, of *C. angustifolia* decreased from 0.55 in the 1970s to 0.50 in the 2000s and that of *G. spiculosa* from 0.30 in the 1970s to 0.13 in the 2000s, accompanied by an increase in mesophytes. Although importance values for both hygrophytes declined, the former decreased by 9%, while the latter decreased 57%. Another sequentially documented *Carex lasiocarpa* community in a hydric habitat showed that the importance value of *G. spiculosa* decreased from 0.19 in the 1970s to 0.13 in the 2000s, while that of *C. angustifolia* increased from 0.021 to 0.023 [40]. Consequently, *C. angustifolia* is more competitive than *G. spiculosa* and *G. spiculosa* is more environmentally sensitive than *C. angustifolia*, which might be partly attributed to the greater acclimated capacity of *C. angustifolia* responding to precipitation and/or temperature change (excluding anthropogenic activities). Firstly, the greatest above-ground biomass of *C. angustifolia* presents in the +4°C treatment (Fig. 6A), while that of *G. spiculosa* is in the +2°C and +4°C manipulations (Fig. 6C), suggesting that *C. angustifolia* might be more acclimated to greater warming, even occasional extreme high temperatures. Secondly, there is no significant difference in the precipitation manipulations for *C. angustifolia*'s leaf area (Table 3) or above-ground biomass (Table 3), while either -50% or +50% of precipitation significantly decreased the leaf area (Fig. 3B) in August and September and above-ground biomass (Fig. 6D) of *G. spiculosa*.

Considering the warming trend of 2.5–5.4°C in the Sanjiang Plain within this century [41], it is predicted that the distribution of *C. angustifolia* will expand while that of *G. spiculosa* will shrink. However, the composition dynamics of these communities will be determined both by the intrinsic biological characteristics of each species and the external environment including climate change [5], [42]. The species-specific responses described in this research coupled with inter-species interactions demonstrate the complexity of multi-factor effects. In addition to climate, other factors, e.g. soil nutrient concentrations, will modify or attenuate temperature and precipitation effects [35]. Consequently, minimization of other external (anthropogenic) factors is required in order to assess climate change impacts and this can best be achieved using well-conserved boreal wetlands.

### Implications for boreal wetland conservation and restoration

Boreal wetland ecosystems, especially those that are well conserved, have an innate resistance and acclimated self-regulation potential, allowing them to respond to climate change and variation in habitat conditions [7], [8]. However, long-term stress caused by high temperature, extreme drought or flooding, or the degradation and eventual loss of dominant species or functional groups will alter fundamental ecosystem properties and processes, because changes in the abundance, production, or distribution of species are often sufficient to alter both structure and function of the ecosystem [43]. Therefore, investigating the thresholds of irreversible climate change effects is both urgent and critical to the conservation and restoration of boreal wetland ecosystems.

Stability and acclimation can by delineated by ecological thresholds. Within these thresholds, the species interactions of freshwater wetland plants tend to shift from competitive to facilitative with increased stress [44]; however, when the environment changes beyond these thresholds, perhaps through a combination of habitat alteration by climate change and direct damage by humans, boreal wetlands may shift to other ecosystems such as grassland or shrubland [45]. This may result in loss of the organic carbon sequestered in these wetlands for hundreds or thousands of years, ultimately reducing the net cooling effect of a carbon sink on global warming [7], [45]. To avoid this consequence, conservation policies and restoration practices will need to be revised in the face of potential thresholds for irreversible change to key wetland plant species under climate change. For example, hydrologically damaged wetlands will experience greater vulnerability to climate change effects compared to wetlands with an intact hydrological regime [8]. According to our results (Fig. 6D), the stability of the natural surface water table is critical for the conservation and restoration of the *G. spiculosa* population, which indicates special actions may be required at both the management and policy level if this species is to be safeguarded in the future.
